# Intriguing Encounter: Retrieval of a Crescent-Shaped Metallic Foreign Body From the Maxillofacial Region

**DOI:** 10.7759/cureus.63098

**Published:** 2024-06-25

**Authors:** Parmarth M Sonpal, Dr Bhushan Mundada, Nitin Bhola, Rajanikanth K, Deepankar Shukla, Sanjana N Wadewale, Riya Goyal

**Affiliations:** 1 Oral and Maxillofacial Surgery, Sharad Pawar Dental College and Hospital, Wardha, IND

**Keywords:** patient, local anaesthesia, injury, malar region, foreign body

## Abstract

This case report presents the clinical details and management of a 40-year-old male welder who presented with a foreign body lodged in his left malar region for eight months following a work-related injury. The patient experienced persistent pain, intermittent swelling, and occasional discharge, prompting medical evaluation. Radiographic imaging confirmed the presence of a metallic object, and surgical exploration under local anesthesia led to successful removal. Postoperatively, the patient experienced complete resolution of symptoms, highlighting the importance of prompt intervention in cases of foreign body impaction to prevent complications and improve patient outcomes.

## Introduction

Foreign bodies embedded in soft tissues represent a common challenge in clinical practice, often necessitating prompt diagnosis and intervention to prevent complications [1]. Occupational hazards, particularly in professions involving exposure to metallic debris, increase the risk of foreign body impaction, as evidenced in this case of a welder sustaining a foreign body in the left malar region [2]. Welders are frequently exposed to various metal particles during their work, predisposing them to traumatic injuries [3]. This report aims to elucidate the clinical presentation, diagnostic workup, and management of such cases, emphasizing the importance of tailored approaches to ensure optimal outcomes. By presenting this case, we underscore the significance of maintaining a high index of suspicion for foreign bodies in occupational settings and highlight the efficacy of surgical exploration under local anesthesia as a safe and effective intervention. This case report contributes to the existing literature on soft tissue foreign bodies, providing insights into their management and reinforcing the need for vigilance in occupational safety practices.

## Case presentation

**Figure 1:**
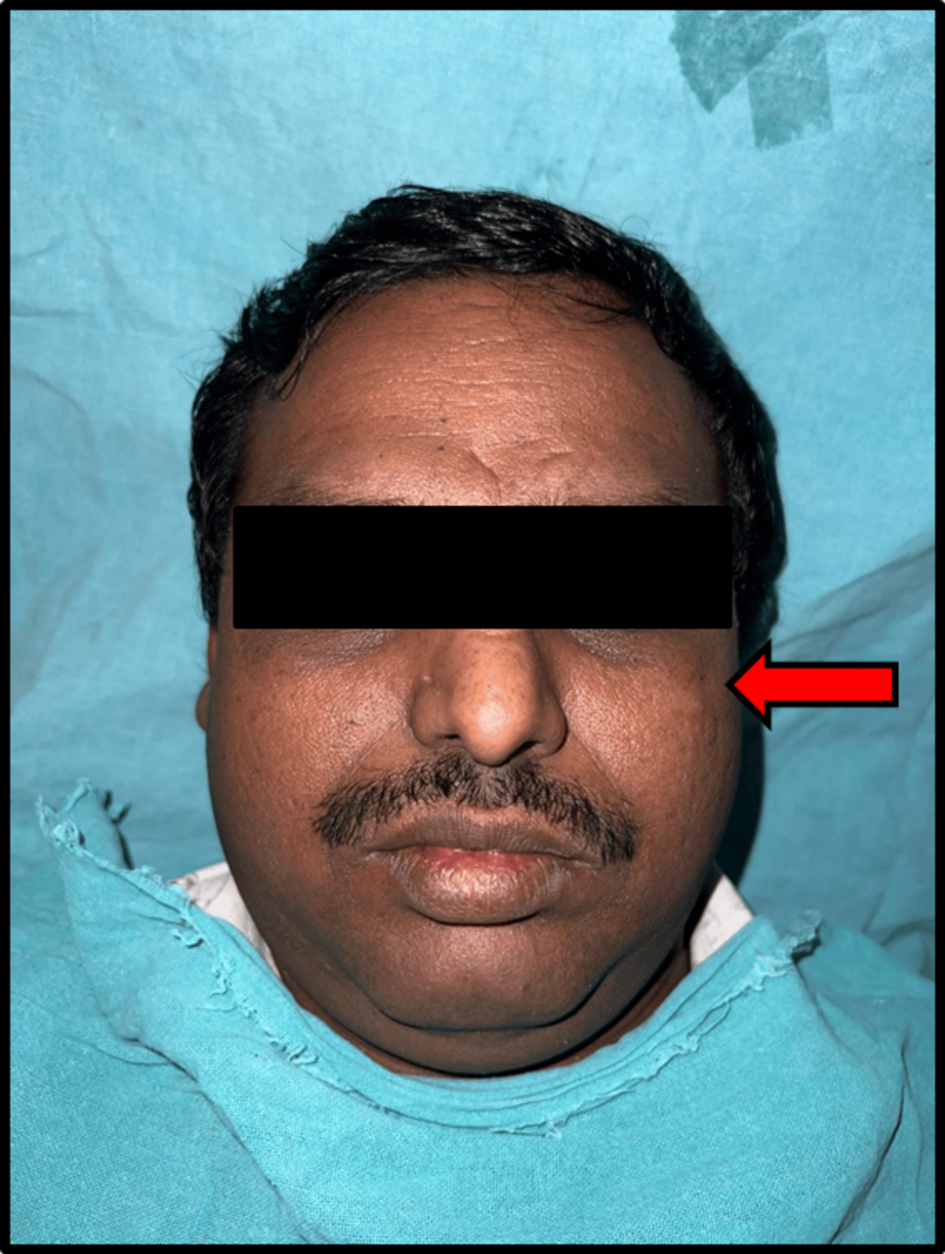
Left malar region showing minimal soft tissue swelling and a hazy scar

The patient, a 40-year-old male welder, presented to the outpatient department of our institute with a chief complaint of persistent pain, intermittent swelling, and occasional discharge from his left malar region, which had been ongoing for eight months (Figure 1).

**Figure 2:**
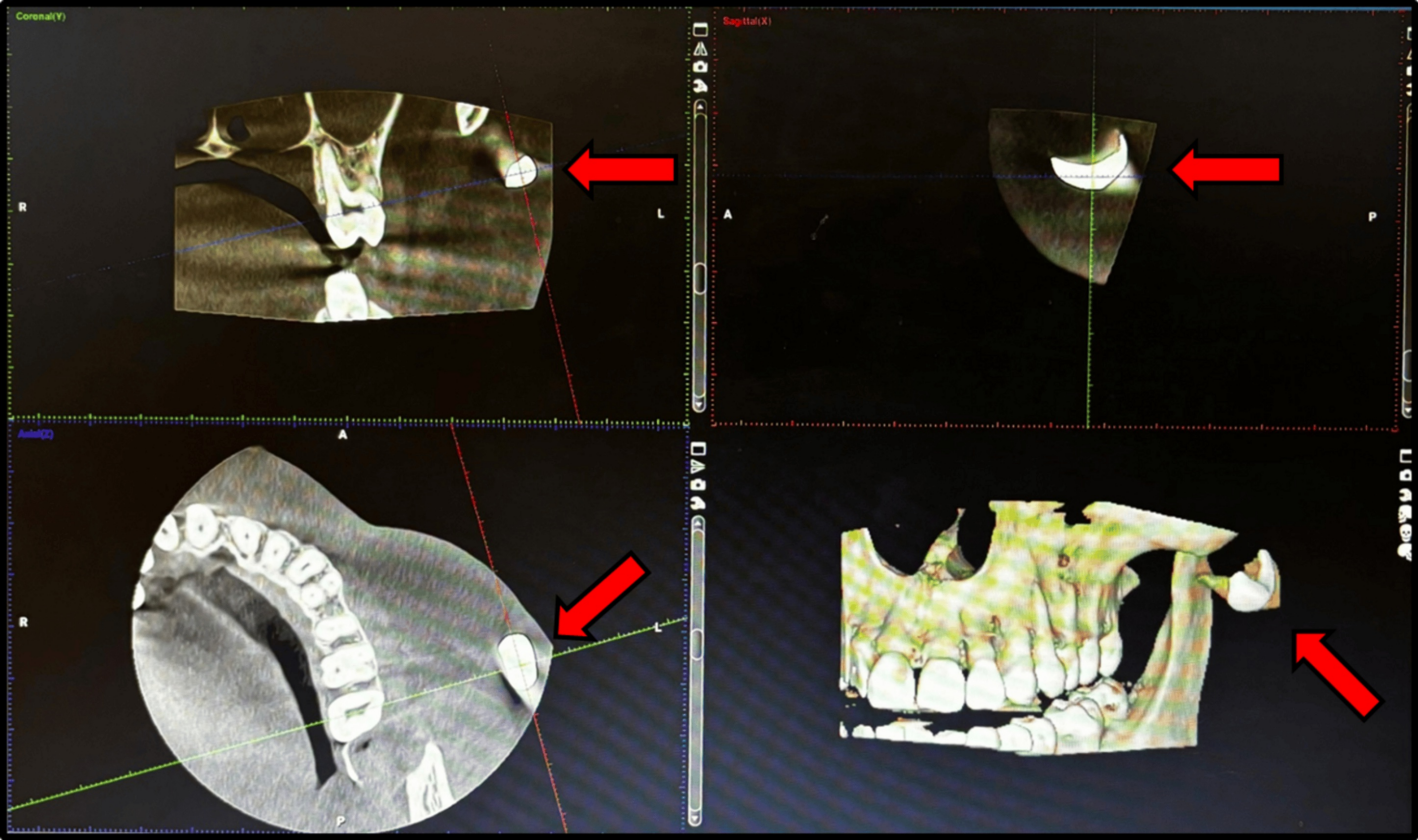
Cone beam computed tomography (CBCT) showing foreign body over left malar region CBCT image in different sections

He reported sustaining a traumatic injury while at work, during which a foreign body had penetrated the soft tissues of his left cheek. Physical examination revealed a palpable mass in the affected area, accompanied by tenderness on palpation. There were no signs of systemic infection or significant soft tissue damage. Radiographic imaging, including cone beam computed tomography (CBCT) scans, confirmed the presence of a metallic object lodged in the soft tissues of the left malar region, prompting surgical intervention (Figure 2).

**Figure 3:**
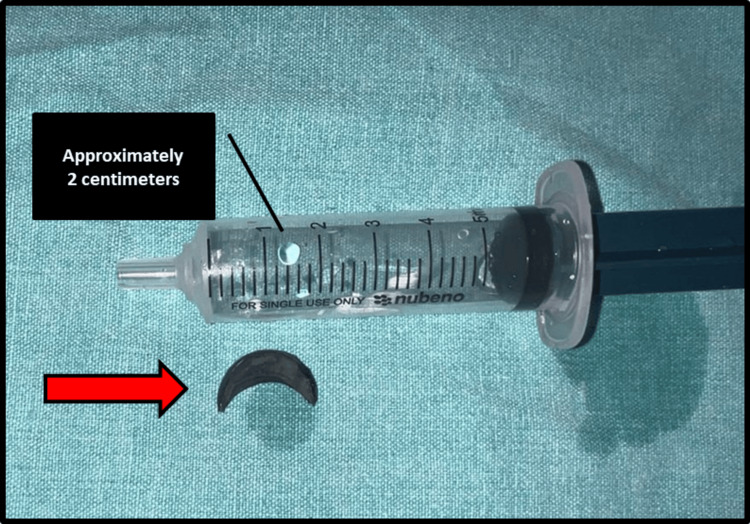
Extracted crescent-shaped metallic foreign body

Under all aseptic precautions, local infiltration was given. After achieving all signs and symptoms of local anesthesia, a stab incision was given through the scar over the left malar region. Exploration was done using artery forceps (Video 1), and the metallic foreign body was removed (Figure 3).

**Video 1:** Exploration and foreign body removal from the malar region

**Figure 4:**
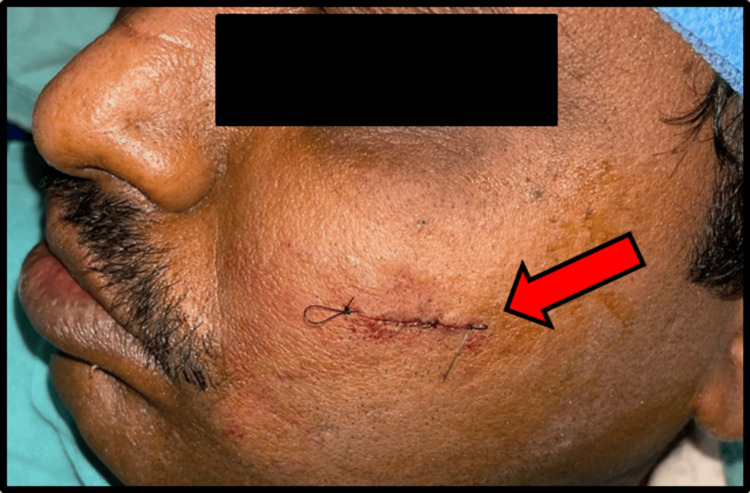
Aesthetic closure of given stab incision using 3-0 Vicryl and 4-0 Ethilon

Hemostasis was achieved, and sub-cuticular sutures were given using 3-0 Vicryl and 4-0 Ethilon (Figure 4).

Postoperatively, antibiotics and analgesics were given for five days.

## Discussion

Foreign bodies in the facial region, particularly in individuals with occupational exposure to metal debris, such as welders, present unique diagnostic and management challenges [4]. In this case, the patient's occupation as a welder placed him at increased risk of sustaining such injuries due to the nature of his work environment. The prolonged duration between the initial injury and presentation to our clinic underscores the importance of heightened awareness among clinicians regarding the potential for foreign body impaction in high-risk populations [5].

Radiographic imaging played a crucial role in confirming the presence and localization of the foreign body, guiding subsequent surgical planning. Surgical exploration under local anesthesia was chosen as the preferred approach, given the superficial location of the foreign body and the absence of significant surrounding tissue involvement [6]. This approach minimized the risk of complications associated with general anesthesia and facilitated a prompt resolution of the patient's symptoms.

The successful removal of the metallic foreign body resulted in immediate relief of the patient's pain and swelling, highlighting the efficacy of surgical intervention in such cases. Postoperative follow-up examinations confirmed the absence of infection or recurrence, with the patient experiencing a complete resolution of symptoms. Furthermore, patient education regarding occupational safety measures aimed at preventing similar incidents in the future was emphasized during the postoperative period.

This case underscores the importance of a multidisciplinary approach to the management of soft tissue foreign bodies, involving close collaboration between clinicians, radiologists, and surgeons. Early recognition and intervention are paramount in preventing complications such as infection, tissue necrosis, and migration of the foreign body. By presenting this case, we aim to contribute to the existing body of literature on the management of soft tissue foreign bodies, particularly in occupational settings, and emphasize the significance of tailored interventions to optimize patient outcomes.

Review of similar cases in literature

A review of the literature reveals several cases similar to ours, highlighting various aspects of diagnosis, management strategies, and outcomes associated with facial soft tissue foreign bodies. Notable cases include the following:

· Melo et al. (2017) - Presented a case where radiographic imaging guided the precise localization and surgical removal of a metallic foreign body in the facial region, similar to our approach [7].

· Khandelwal et al. (2018) - Reported a case of a welder with a superficial foreign body in the maxillary soft tissues, emphasizing occupational hazards and diagnostic challenges [4].

· Alrasheed et al. (2021) - Discussed the efficacy of local anesthesia for surgical exploration and removal of superficial facial foreign bodies, supporting our decision-making process [8].

These cases collectively reinforce the significance of prompt recognition, appropriate imaging modalities, and tailored surgical interventions in managing facial soft tissue foreign bodies, particularly in occupational settings. They underscore the diverse presentations and challenges encountered, thereby enriching our understanding and guiding clinical practice.

## Conclusions

This case report highlights the clinical presentation, diagnostic workup, and successful management of a 40-year-old male welder who presented with a foreign body lodged in his left malar region following a work-related injury. The prompt identification and surgical removal of the metallic foreign body under local anesthesia resulted in immediate symptom relief and a favorable outcome for the patient. Through this case, we underscore the importance of maintaining a high index of suspicion for foreign bodies in occupational settings, particularly among individuals with exposure to metal debris. Early intervention is crucial to prevent complications and ensure optimal patient outcomes. Additionally, patient education on occupational safety measures is essential in preventing similar incidents in the future. Integrating our case with similar reported cases contributes to the cumulative knowledge base, emphasizing the importance of a systematic approach and multidisciplinary collaboration in optimizing outcomes for patients with similar clinical presentations.
